# Research on the Blast Mitigation Performance of Polyurea–Steel Composite Plates Based on Constrained Layer Damping Structures

**DOI:** 10.3390/polym17182461

**Published:** 2025-09-11

**Authors:** Rui Zhang, Qi Dong, Zhiqiang Fang, Yongjun Deng, Pengcheng Li, Hao Xu, Weibo Huang

**Affiliations:** 1 Shock and Vibration of Engineering Materials and Structures Key Lab of Sichuan Province, Southwest University of Science and Technology, Mianyang 621010, China; zhangray126@126.com (R.Z.); dyj820@swust.edu.cn (Y.D.); 2 Institute of Chemical Materials, China Academy of Engineering Physics, Mianyang 621999, China; xuhaogg123@163.com; 3 Qingdao Shamu Advanced Material Co., Ltd., Qingdao 266108, China; fangzhiqiang1015@163.com (Z.F.); spua@163.com (W.H.); 4 School of Civil Engineering and Architecture, Southwest University of Science and Technology, Mianyang 621000, China; njustlpc@163.com; 5 School of Civil Engineering, Qingdao University of Technology, Qingdao 266525, China

**Keywords:** polyurea, damping, blast mitigation, constrained layer damping

## Abstract

To address the challenge of balancing the damping performance with mechanical strength in conventional polyurea materials for blast mitigation, this study develops a constrained layer damping coating structure using Q413t viscoelastic polyurea (Q413t) as the damping layer and FPU-1 flexible polyurea (FPU-1) as the constraining layer. The mechanical behaviors of both types of polyurea were characterized through tensile testing at varying loading speeds, while dynamic thermomechanical analysis was utilized to evaluate their damping properties. A 75 g TNT contact explosion test and finite element simulation were employed to explore the protective mechanism. The results show that Q413t demonstrates significant strain-rate sensitivity under intermediate-strain-rate conditions, whereas FPU-1 exhibits minimal variation in mechanical strength. Q413t demonstrates a superior damping performance over a frequency range of 0–10^4^ Hz. FPU-1 achieved a loss factor of 0.3 when the loading frequency reached 10^4^–10^5^ Hz. Under the 75 g TNT contact explosion load, the configuration with a 1 mm damping layer and a 3 mm constraint layer achieved a maximum displacement reduction of 35.26%. In the constrained layer damping coating, the damping layer contributes to blast protection through energy dissipation and load distribution, while the constraining layer reduces structural deformation by limiting displacement. Relative motion between the layers further enhances the overall damping performance. The constrained layer damping coating provides optimal blast protection when the damping-to-constraining layer thickness ratio is 1:3. The constrained layer damping coating enables the synergistic optimization of mechanical strength and energy dissipation, effectively mitigating structural deformation induced by blast loading and demonstrating promising engineering application potential.

## 1. Introduction

Blast-induced structural damage and secondary injuries caused by ejected fragments present a critical threat to personnel safety and essential infrastructure [1,2]. Polyurea has emerged as a high-performance elastomeric material, exhibiting excellent blast mitigation, impact resistance, and damping capabilities [3,4,5,6,7], and has therefore become a focal point in explosion protection research. When exposed to blast loading, metallic structures may generate high-velocity fragments that can cause extensive damage to surrounding components. The existing studies indicate that polyurea can absorb shockwave energy through large deformations, effectively reducing the dynamic responses of structures and inhibiting fragment scattering [8]. As a result, polyurea-coated metallic structures have been widely recognized as a key direction in the development of advanced blast-resistant structural systems.

Recent years have witnessed growing research attention toward polyurea-coated metallic structures in the field of blast mitigation [9,10,11]. Zhang et al. [12] conducted a series of explosion experiments to compare the energy absorption and protective mechanisms of two types of polyurea elastomers with distinct mechanical properties (high hardness and high ductility) when applied to different spray-coated positions. The results demonstrate a superior blast mitigation performance when high-ductility polyurea is coated onto the back surfaces of steel plates. He et al. [13] investigated the blast resistance of polyurea-coated armor composite structures through near-field air explosion tests using equivalent TNT charges. The blast mitigation performance of polyurea was observed to manifest as energy absorption/storage during the tensile phase and as energy release/dissipation in the rebound phase, while the magnitudes of the tensile and rebound deformation correlated with the blast loading and interfacial bond strength. Researchers have investigated polyurea formulation refinement and multilayer structural design optimization for polyurea-coated metallic composite structures to further enhance the protective performance of polyurea [14,15,16]. However, the current research shows limitations. Primarily, most of the current studies address only the passive protection (structural deformation constraint) of polyurea while overlooking the dynamic interactions at coating–metal interfaces under blast loading. Moreover, the existing research predominantly employs homogeneous polyurea and focuses on its protective performance against blast [17] and impact loading [7,18], with insufficient investigation into the microstructural modulation of polyurea and its damping performance.

The molecular chain segment structure of polyurea dictates its exceptional damping properties, and the role of these properties in blast resistance has garnered increasing research attention in recent years [19,20]. Polyurea with high-damping properties can effectively convert impact kinetic energy into heat through the relaxation and viscoelastic dissipation of the internal molecular chain segments of the polyurea, thereby reducing the strain rates and peak stresses in steel plates [12]. The enhanced damping properties of polyurea significantly increase the deformation resistance in steel plates while delaying yield failure initiation under blast loading. However, the consideration of the damping properties in structures involves trade-offs: excessively high damping levels may diminish material stiffness and compromise the overall structural load-bearing capacity. Consequently, balancing the damping performance and stiffness through formulation design or structural optimization while maintaining the mechanical properties of polyurea constitutes the key approach for enhancing its blast resistance performance [11,20]. Nevertheless, the current research remains limited, particularly in terms of the systematic analysis of the correlative mechanisms between damping regulation and protective performance.

To overcome the limitations in the blast protection performance of single-type polyurea coatings observed in the current studies, and building upon our previous research on the blast mitigation mechanisms of damping polyurea identified in the current research [20], this study proposes a constrained layer damping coating structure by integrating flexible polyurea (FPU-1) and viscoelastic damping polyurea (Q413t). The design leverages the principles of constrained layer damping to enhance energy dissipation and structural protection under blast loading. The constrained layer damping coating was integrated with steel plates to improve their blast resistance, resulting in a composite structure that is hereinafter referred to as the polyurea–steel composite plate. In this polyurea–steel composite plate, the viscoelastic damping polyurea layer with a high loss factor is applied to the steel plate surface to dissipate impact energy, while the flexible polyurea is coated on the outer surface to constrain the structural deformation. This configuration enables a synergistic optimization between the coating strength and energy dissipation capacity, aiming for a substantial improvement in the blast mitigation of steel plates. By combining mechanical testing under varying strain rates, dynamic thermomechanical analysis (DMA), explosion tests, and finite element simulations, the dynamic response and energy dissipation mechanisms of the constrained layer damping coating were analyzed. These findings contribute to the advancement of design theory and technical support for blast-mitigation applications involving polyurea-coated metallic structures.

## 2. Test Specimen Preparation Process

### 2.1. Polyurea

The main components of Q413t and FPU-1 synthesized in this study are presented in Table 1.

The A component of FPU-1 consists of a mixture of MDI-50 and its prepolymer, while the B component primarily comprises the end amino polyether P1000 and the amine-based chain extender ML-400. Prior to synthesis, the A and B components of FPU-1 must be separately and thoroughly mixed under nitrogen protection. Both FPU-1 and Q413t are fabricated using a high-pressure spray process with a volumetric mixing ratio of 1:1 between the A and B components. Before spraying, the equipment and raw materials are preheated to maintain a reaction temperature of 65 °C. During spraying, the system pressure is maintained at 17.24 MPa. Once spraying is complete and the sample surface has dried, the samples are cured under controlled conditions of 23 ± 2 °C and 60 ± 15% relative humidity for 7 days.

### 2.2. Preparation Process of Polyurea–Steel Composite Plate

The polyurea–steel composite plate employs the Q235 steel plate as the protective substrate material. The test utilized the steel plate with dimensions of 300 mm (length), 300 mm (width), and 10 mm (height). In the constrained-layer-damping coating structure, Q413t functions as the damping layer, while FPU-1 acts as the constraining layer. To investigate how varying coating structure ratios affect the explosion protection performance of steel plates, this study employs three distinct coating configurations to evaluate the blast resistance of polyurea–steel composite plates under different structural designs. The specific dimensions and schematic diagrams of each polyurea–steel composite plate are shown in Table 2.

**Figure 1:**
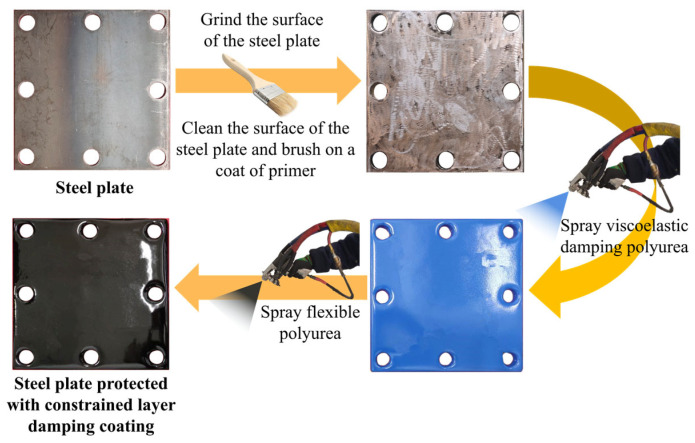
Schematic diagram of the preparation process for polyurea–steel composite plates.

The preparation process of the polyurea–steel composite structure is illustrated in Figure 1. First, the steel plate surface is ground to promote adhesion by increasing roughness. Following grinding, the steel plate surface is thoroughly cleaned, and an epoxy-modified polyurethane primer is applied to improve the adhesion between the coating and the substrate. Once the primer layer has reached surface dryness, the polyurea damping layer is sprayed using a high-pressure spraying system. After the damping layer has surface-dried, the flexible polyurea coating is applied using the same spraying equipment. Upon completion of spraying, the fabricated polyurea–metal composite plate is cured under the same environmental conditions specified in Section 2.1 for a duration of 7 days.

## 3. Experiments

### 3.1. Mechanical Performance Test

The mechanical properties of polyurea under low strain rates were evaluated in accordance with GB/T 528-2009 (rubber, vulcanized, or thermoplastic determination of tensile stress–strain properties), using a universal testing machine. Low-strain-rate tests were conducted at loading speeds of 1.5 mm·min^−1^ (2.5 × 10^−5^ m·s^−1^), 15 mm·min^−1^ (2.5 × 10^−4^ m·s^−1^), and 150 mm·min^−1^ (2.5 × 10^−3^ m·s^−1^). For the intermediate-strain-rate range, mechanical tests were performed using a high-speed testing machine at loading speeds of 1 m·s^−1^, 5 m·s^−1^, 12 m·s^−1^, and 15 m·s^−1^.

### 3.2. Dynamic Mechanical Analysis

The DMA of polyurea was carried out at Southwest University of Science and Technology in accordance with GJB 981A-2021 (forced nonresonant dynamic testing method for viscoelastic-damping materials), using a dynamic mechanical analyzer in three-point bending deformation mode. Based on the glass transition temperature (*T*_g_) of polyurea and the temperature ranges of the rubbery states reported in the Refs. [2,19,20], the experimental temperature range of −70 °C to 90 °C was selected in this study, along with test frequencies of 1 Hz, 25 Hz, and 50 Hz.

### 3.3. Explosion Test

To investigate the deformation behavior of polyurea–steel composite plates under blast loading and their effectiveness at providing blast protection to metallic structures, contact explosion tests were conducted on the polyurea–steel composite plates with the varying coating configurations discussed in Section 2.2, using a 75 g TNT charge.

**Figure 2:**
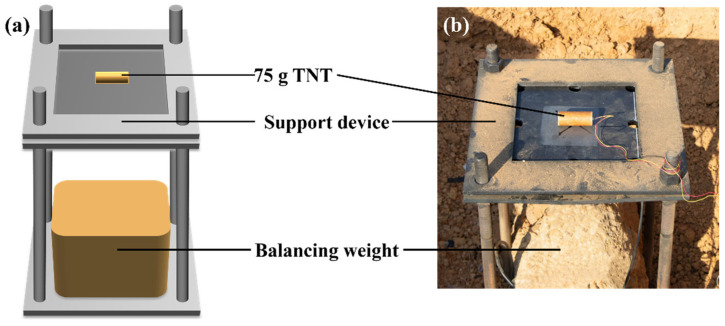
Explosion test device setup: (**a**) schematic of testing arrangement; (**b**) practical test setup.

The support and loading configuration for the polyurea–steel composite plate is illustrated in Figure 2. Prior to the explosion test, a pit 1.5 m in depth was excavated, and the support device was placed inside to ensure horizontal alignment. A balancing weight was positioned beneath the support device to maintain stability during the explosion, preventing overturning. The edges of the excavation pit were raised above the support structure to contain any fragments and ensure the safety of surrounding personnel. The polyurea–steel composite plate was supported from below using an intermediate skeletonized steel plate and constrained from above using a steel plate of identical specifications. This support device provides simply supported boundary conditions through the lower steel plate, while the upper steel plate effectively restrains the specimen from upward displacement during and after the explosion. This dual-function design not only maintains the structural stability of the specimen under blast loading but also contains specimen ejection, thereby significantly improving the test safety. A 75 g cylindrical TNT charge was positioned at the geometric center of the specimen for the purpose of loading.

## 4. Experimental Results and Analysis

### 4.1. Mechanical Properties Under Different Strain Rates

**Figure 3:**
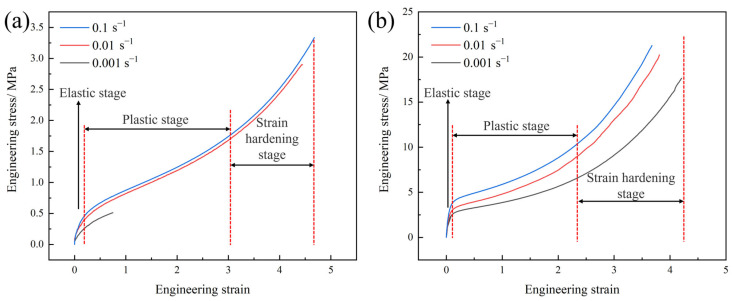
*σ–ε* curves of polyurea at low strain rates: (**a**) Q413t; (**b**) FPU-1.

Previous studies have shown that the strain-rate sensitivity of polyurea is a critical characteristic that distinguishes it from other materials commonly employed in blast mitigation applications [21,22]. The mechanical response of polyurea under different strain rates is a crucial factor in evaluating its effectiveness in blast mitigation. First, the mechanical properties of two types of polyurea under low-strain-rate conditions were analyzed. Figure 3 shows the engineering stress–engineering strain (*σ–ε*) curves of Q413t and FPU-1 in the low-strain-rate-loading condition.

The *σ–ε* curves of FPU-1 and Q413t under low-strain-rate conditions reveal three distinct deformation stages: the elastic stage, the plastic stage, and the strain-hardening stage. In the elastic stage, both materials exhibited a limited strain range, spanning from 0 to 0.13. During the plastic deformation stage, Q413t displayed a strain range of 0.13 to 3.07, while FPU-1 exhibited a slightly narrower range of 0.13 to 2.36. Q413t exhibited a less pronounced change in the slope of its *σ–ε* curve during the strain-hardening stage compared to FPU-1. To further examine the strain-rate sensitivity of these two polyurea materials under low-strain-rate loading, their modulus values and key mechanical performance parameters were analyzed, as presented in Table 3.

As shown in Table 3, when the strain rate increases from 0.001 s^−1^ to 0.1 s^−1^, the elastic modulus of Q413t increases by 72%, while that of FPU-1 increases by 51%. However, regarding the tangent modulus, Figure 3 clearly indicates that Q413t shows minimal variation, whereas FPU-1 demonstrates a notable increase. Specifically, the tangent modulus of FPU-1 rises by 66% under the same strain-rate increment. Under the influence of the strain-rate effect, the tensile strengths of both types of polyurea increase with the rising strain rate, while the breaking elongation remains relatively stable without a significant decrease. Notably, the *σ–ε* curve of Q413t at a strain rate of 0.001 s^−1^ reflects relatively poor breaking elongation and mechanical strength. This phenomenon can be attributed to the higher internal bubble content in Q413t. At low strain rates, the loading process exhibits a slow deformation rate, which can be approximated as a constant extension (static holding) loading mode. Once the coating cracks propagate to the internal bubble regions, the prolonged tensile stress accelerates the rapid failure of the coating.

**Figure 4:**
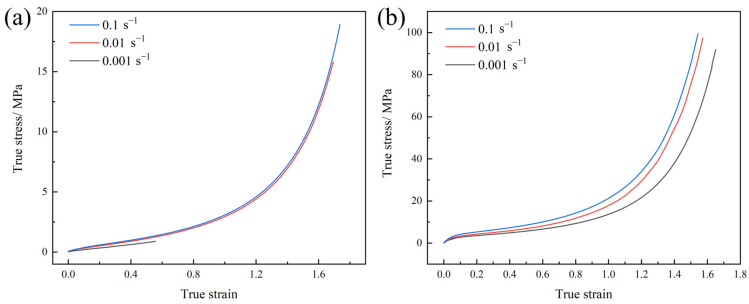
*σ*_T_*–ε*_r_ curves of polyurea at low strain rates: (**a**) Q413t; (**b**) FPU-1.

The existing research demonstrates that the necking phenomenon in polyurea under tensile deformation significantly influences its mechanical behavior [20]. From a practical engineering perspective, the tensile deformation process of coated protective structures involves not only coating failure at defect sites under external loads but also a reduction in the coating thickness during deformation. Utilizing both σ-ε and true stress–true strain (*σ*_T_–*ε*_r_) curves to analyze polyurea enables a more comprehensive evaluation of its strain-rate effect. Therefore, the *σ–ε* curves of both materials were converted into *σ*_T_*–ε*_r_ curves (Figure 4) for more accurate analysis.

The *σ*_T_*–ε*_r_ curves of the polyurea exhibit a clear bilinear characteristic, indicating the absence of a well-defined elastic stage. From a mechanical strength perspective, at a strain rate of 0.1 s^−1^, the maximum true stresses of Q413t and FPU-1 reached 18.95 MPa and 99.52 MPa, respectively. This indicates that at the point of fracture, the stress experienced by the tensile fracture surface of high-elongation polyurea was significantly higher than the corresponding engineering stress. Regarding the strain-rate effect, when the strain rate increased from 0.01 s^−1^ to 0.1 s^−1^, the maximum true stresses of Q413t and FPU-1 increased by only 16% and 2%, respectively. A comparison of the elastic modulus, tangent modulus, and tensile strength shows that both types of polyurea exhibit relatively low strain-rate sensitivity under low strain rates.

**Figure 5:**
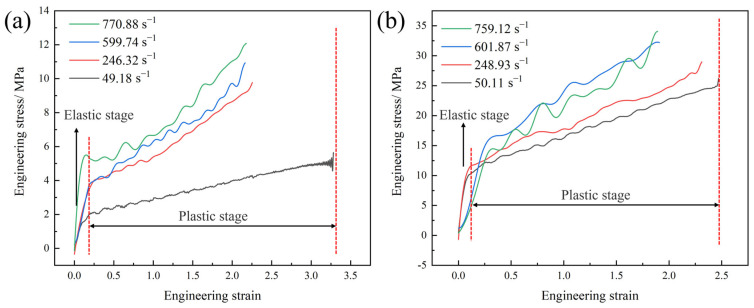
*σ–ε* curves of polyureas at intermediate strain rates: (**a**) Q413t; (**b**) FPU-1.

To further investigate the mechanical behaviors of the two types of polyurea under high-speed loading, their mechanical properties at intermediate strain rates were examined. Figure 5 presents the *σ*–*ε* curves of Q413t and FPU-1 under intermediate-strain-rate conditions.

Compared to the *σ*–*ε* curves at low strain rates, those of the two types of polyurea at intermediate strain rates exhibit notable differences. The *σ-ε* curves of the two types of polyurea at intermediate strain rates exhibit clear bilinear behavior, consisting solely of an elastic stage followed by a plastic stage. During the elastic stage, the elastic modulus of Q413t increased markedly from 11.17 MPa at a strain rate of 49.18 s^−1^ to 30.51 MPa at 770.88 s^−1^, representing a 173% increase under intermediate-strain-rate conditions. In contrast, FPU-1 exhibited elastic moduli of 66.74 MPa and 58.51 MPa at comparable strain rates of 50.11 s^−1^ and 759.12 s^−1^, respectively, indicating no significant strain-rate enhancement. In terms of the tangent moduli, the tangent moduli of Q413t at strain rates of 49.18 s^−1^ and 770.88 s^−1^ were 1.04 MPa and 3.62 MPa, respectively, an increase of 248%. For FPU-1, at strain rates of 50.11 s^−1^ and 759.12 s^−1^, the corresponding tangent moduli were 5.97 MPa and 10.41 MPa, respectively, with a 74% increase in the tangent modulus. It can also be clearly seen from Figure 5 that both Q413t and FPU-1 significantly improved in tensile strength. However, it should be noted that due to the strain-rate effect, the breaking elongation of the polyurea also decreased simultaneously. When comparing the breaking elongation at strain rates of approximately 50 s^−1^ and 760 s^−1^, Q413t and FPU-1 exhibit reductions of 111% and 58%, respectively. This reduction in the breaking elongation leads to the maximum true stress of polyurea tending to stabilize or even decrease, with the failure behavior manifesting as brittle fracture. Furthermore, in terms of the curve characteristics, the σ-ε curves of the two types of polyureas exhibit clear fluctuations, contrasting with the smooth curves observed at low strain rates. This phenomenon can be attributed to the oscillation of the σ-ε curve, which was caused by the relatively low mechanical strength of polyurea in comparison to the load-bearing capacity of the testing equipment, as observed in the tensile behavior studies conducted by Cui et al. [21].

**Figure 6:**
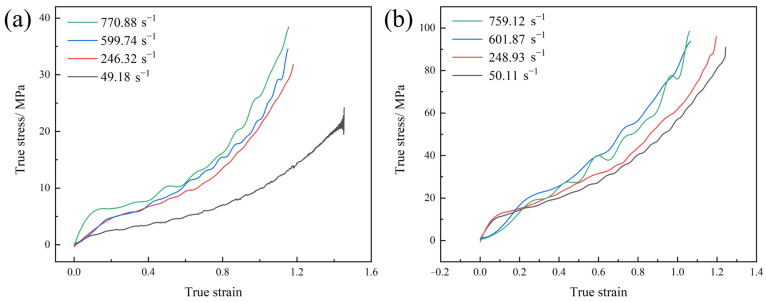
*σ*_T_–*ε*_r_ curves of polyurea at intermediate strain rates: (**a**) Q413t; (**b**) FPU-1.

Figure 6 shows the *σ*_T_–*ε*_r_ curves of Q413t and FPU-1 under intermediate-strain-rate conditions. Q413t exhibits a significant increase in maximum true stress with the increasing strain rate. At 770.88 s^−1^, its maximum true stress reaches 38.64 MPa—a 104% increase compared to that at 0.1 s^−1^. In contrast, FPU-1 exhibits a pronounced strain-rate dependence in terms of its breaking elongation. When the strain rate increases to 759.12 s^−1^, the breaking elongation of FPU-1 decreases to 189%, a 49% reduction relative to the value at 0.1 s^−1^. However, the tensile strength of FPU-1 remains relatively unchanged with the increasing strain rate. Consequently, unlike Q413t, FPU-1 does not show a notable increase in maximum true stress. At 759.12 s^−1^, the maximum true stress of FPU-1 is only 98.31 MPa, nearly identical to that at 0.1 s^−1^ (see Figure 4b).

Thus, in the context of high-speed load protection, the strain-rate effect can enhance the material strength. However, for polyurea, where the breaking elongation is significantly reduced under high strain rates, the maximum true stress may also decrease. This reduction in true stress can considerably impair the protective performance of polyurea coatings against high-speed loads and may even lead to more severe structural damage due to the brittle fracture of the coating.

### 4.2. Dynamic Thermomechanical Analysis

#### 4.2.1. Storage Modulus and Loss Modulus

**Figure 7:**
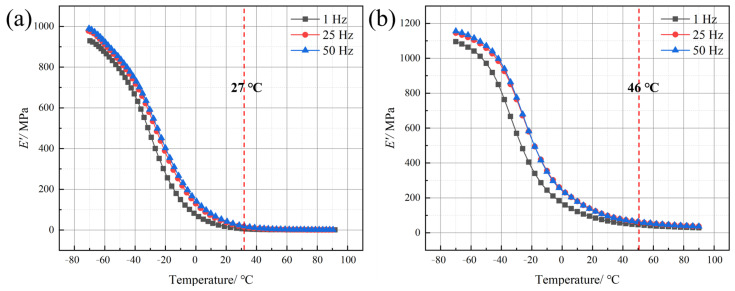
*E′*–T curves of (**a**) Q413t and (**b**) FPU-1 at different loading frequencies.

**Figure 8:**
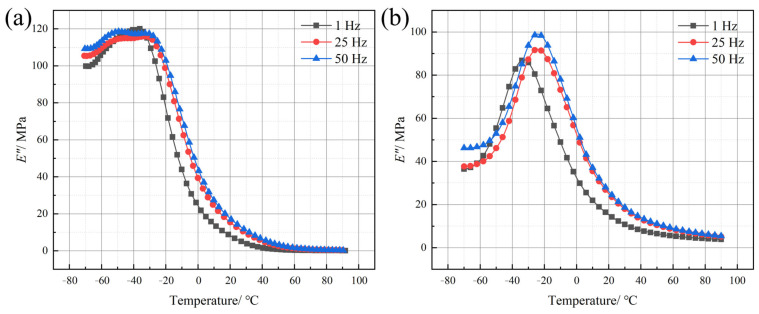
*E″*–T curves of (**a**) Q413t and (**b**) FPU-1 at different loading frequencies.

DMA was performed to assess the damping behavior of polyurea and its response to different loading frequencies. The storage modulus–temperature (*E′*–T) and loss modulus–temperature (*E″*–T) curves of Q413t and FPU-1 at loading frequencies of 1 Hz, 25 Hz, and 50 Hz are shown in Figure 7 and Figure 8, respectively.

From the *E′*–T curves of Q413t and FPU-1, it is evident that within the experimental temperature range (−70 °C to 90 °C), the *E′* values of both types of polyurea gradually decrease from their maximum values with increasing temperature and eventually stabilize. This suggests that the critical temperatures marking the onset of the glassy state for both materials are below −70 °C. Consequently, the *E′*–T curves can be divided into two distinct regions: the transition region and the rubbery plateau region. The critical temperatures separating these regions are 27 °C for Q413t and 46 °C for FPU-1, indicating that FPU-1 exhibits a broader transition temperature range. In terms of the *E′*, the peak values (*E′*_max_) of Q413t and FPU-1 at a loading frequency of 50 Hz are 989 MPa and 1156 MPa, respectively, indicating that the *E′*_max_ of FPU-1 is significantly higher than that of Q413t. Furthermore, under identical temperature conditions, both materials exhibit an increase in the *E′* as the loading frequency rises. These observations align with the mechanical behavior of Q413t and FPU-1 detailed in Section 4.1, as well as with the strain-rate-dependent trends in their mechanical properties, demonstrating a strong correlation between the *E′* and mechanical strength in polyurea.

From the perspective of the mechanical properties and molecular structure, Q413t possesses a higher soft-segment content than FPU-1, resulting in its lower mechanical strength and greater breaking elongation. Previous studies have shown that the soft segments in polyurea exhibit high chain mobility and are highly sensitive to temperature variations [20]. With increasing temperature, the enhanced mobility of these soft segments thereby reduces the structural regularity of the polyurea molecular chains, resulting in a noticeable degradation in mechanical performance. Therefore, during the temperature elevation process, the *E′* of Q413t decreases more rapidly, which consequently results in a significantly narrower transition temperature range for Q413t compared to that for FPU-1.

The loss modulus (*E″*) of polyurea is also notably influenced by the molecular chain segments. As shown in Figure 8, for the *E″*, Q413t, which contains a relatively high proportion of soft segments, can maintain intermolecular sliding even in the low-temperature region, thereby preserving a relatively high *E″.* As the temperature increases, the molecular mobility rises further, and the *E″* of Q413t peaks at −40 °C. Beyond this point, with further temperature elevation, the *E″* decreases sharply due to enhanced segmental motion and increased relative movement among chains connected to soft segments. In contrast, FPU-1, with lower soft-segment content, exhibits restricted molecular chain mobility and limited inter-segment movement at low temperatures. Consequently, the *E″* of FPU-1 in the low-temperature region is significantly lower than that of Q413t. However, this structural characteristic also results in a slower rate of *E″* reduction in FPU-1 compared to that in Q413t.

#### 4.2.2. Loss Factor and Its Master Curve

**Figure 9:**
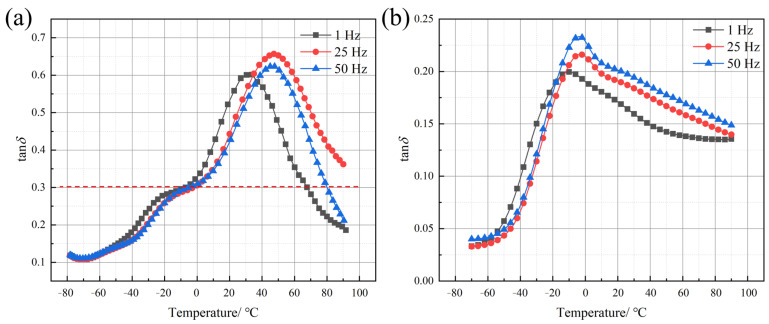
tan*δ*–T curves of (**a**) Q413t and (**b**) FPU-1 at different loading frequencies.

To facilitate a direct analysis of the damping performances of the two types of polyureas, the loss factor–temperature (tan*δ*–T) curves of Q413t and FPU-1 were calculated based on the measured *E′* and *E″*, and the results are presented in Figure 9.

**Figure 10:**
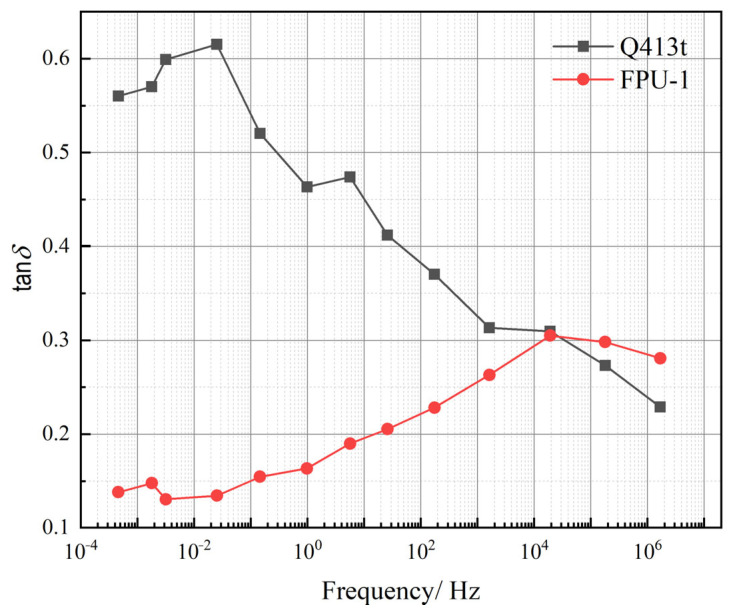
The tan*δ* master curve for each polyurea over a wide frequency domain.

The loss factor (tan*δ*) initially increases and subsequently decreases with rising temperature. The *T*_g_ values of Q413t and FPU-1 are 45 °C and −10 °C, respectively. For Q413t, the tan*δ* decreases rapidly once the temperature exceeds the *T*_g_, which—together with the temperature-dependent variations in the *E′* and *E″* discussed in Section 4.2.1—can be attributed to excessive soft-segment mobility due to rapid material softening above the *T*_g_. In contrast, FPU-1 features a higher hard-segment content, which continues to provide structural support above the *T*_g_, leading to a more gradual decrease in the tan*δ*. However, it is important to note that although FPU-1 exhibits a relatively stable damping performance, its tan*δ* values remain below the threshold for effective damping (tan*δ* ≥ 0.3) across the entire temperature range. In contrast, Q413t, as a damping layer, demonstrates an effective-damping temperature range of −7 °C to 79 °C, which satisfies the required protective conditions. To further investigate the damping behaviors of both types of polyurea under high-frequency loading, the tan*δ* master curves were constructed based on the time–temperature superposition with a reference temperature of 20 °C. The resulting tan*δ* master curves for both materials are presented in Figure 10.

Overall, as indicated by the tan*δ* master curves, both Q413t and FPU-1 exhibit a trend in which tan*δ* first increases and then decreases with increasing loading frequency. A notable difference, however, lies in the frequencies and magnitudes of their peak values: Q413t reaches a maximum tanδ of 0.62 at a low frequency of 0.02 Hz, while FPU-1 attains a peak tanδ of only 0.31 at a much higher frequency of 20,277 Hz. In terms of magnitude, the overall tan*δ* of Q413t is significantly higher than that of FPU-1, suggesting a superior damping performance.

Regarding the effective-damping frequency range, FPU-1 exhibits relatively low tan*δ* values, with effective damping confined narrowly to 10^4^–10^5^ Hz. Under explosive loading conditions, both the structural strain response and the external load itself typically span a broad frequency range. In contrast, Q413t provides effective damping over a frequency range from nearly 0 to 10^4^ Hz, making it suitable for energy absorption under broad-frequency loading. Although Q413t alone does not offer effective damping above 10^4^ Hz, its combination with FPU-1 in a constrained-layer-damping coating structure extends the overall effective frequency range. This synergistic effect enhances the energy absorption capacity and improves protection against high-rate mechanical loads.

### 4.3. Blast Mitigation Performance

**Figure 11:**
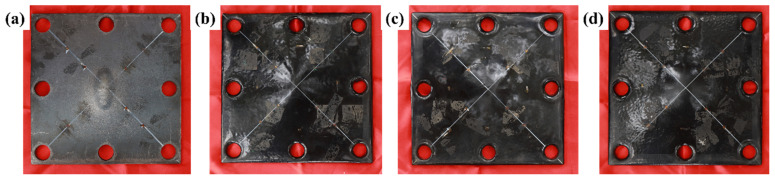
Macroscopic deformation of the back blast face of the plates: (**a**) UC; (**b**) CLD1; (**c**) CLD2; (**d**) CLD3.

To assess the blast resistance of steel plates coated with different constrained layer damping composites, contact explosion tests were conducted using 75 g TNT charges on both bare and polyurea-coated steel plates. The results are summarized in Figure 11.

Macroscopic deformation observations of the back blast faces of the unprotected steel plate and various polyurea–steel composite plates demonstrate that the constrained layer damping coating provides effective explosion protection for the steel plate. This protective performance is primarily manifested through alterations in the load application characteristics and the resulting macroscopic deformation of the protected structure. For the unprotected steel plate subjected to a 75 g TNT contact explosion load, a charge-shaped protrusion developed at the center of the back blast face, located directly beneath the explosive charge (Figure 11a). The protrusion was significantly elevated relative to the surrounding plate surface, with a vertical distance of 0.65 cm between the apex and the edge of the protrusion. This indicates that, in the absence of protective coating, the explosion induced a concentrated load at the point of impact, leading to substantial localized displacement.

In contrast, no evident stress concentration was observed on the back surfaces of the three polyurea–steel composite plates. The polyurea coating effectively redistributed the stress from the contact explosion into uniform plate deformation, thereby altering the load distribution characteristics. This protective mechanism significantly reduced the macroscopic structural damage caused by the explosion. Furthermore, examination of the coating adhesion revealed that all the constrained layer damping composite coatings remained fully intact on the steel plate surface. No interfacial delamination or adhesion failure was observed between the steel plate and Q413t or between Q413t and FPU-1.

**Figure 12:**
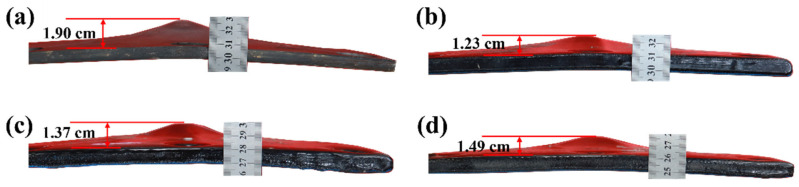
Macroscopic deformation of the side faces of the plates: (**a**) UC; (**b**) CLD1; (**c**) CLD2; (**d**) CLD3.

Regarding structural deformation, the longitudinal deformation of the polyurea–steel composite plates is markedly lower than that of the unprotected steel plate. To visually assess this deformation, measurements were taken along the side profiles of each structure after the explosion. The resulting macroscopic deformation profiles are illustrated in Figure 12.

Observations of the lateral macroscopic deformation clearly indicate that the back blast face of the unprotected steel plate underwent a displacement of 1.9 cm under the explosion load. In contrast, steel plates protected by the constrained layer damping coating exhibit significantly reduced longitudinal displacements. Specifically, compared to the unprotected steel plate, the longitudinal displacements of CLD1, CLD2, and CLD3 are reduced by 35.26%, 27.89%, and 21.58%, respectively, compared to that of the uncoated plate. From the perspective of the lateral deformation amplitude, the polyurea–steel composite plates display relatively mild and uniform deformation patterns. According to the current deformation analysis, the CLD1 configuration, which consists of a 1 mm Q413t layer and a 3 mm FPU-1 layer, offers the most effective protection among the tested composite structures.

## 5. Simulation Model Establishment and Parameter Setting

### 5.1. Establishment of Simulation Model

**Figure 13:**
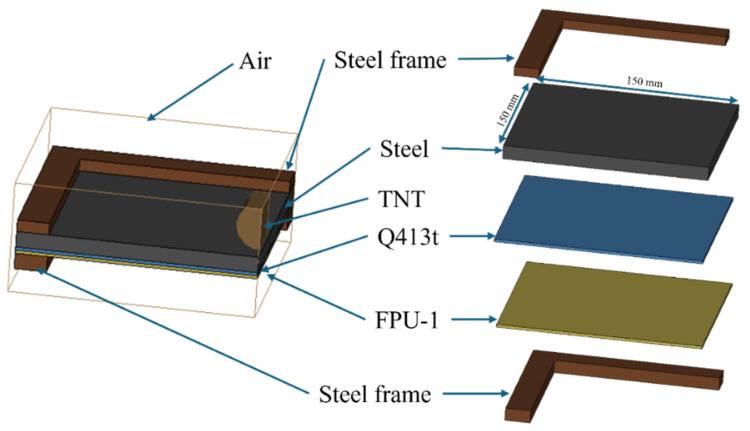
Diagram of finite element model.

To further elucidate the dynamic response and protective mechanisms of polyurea–steel composite plates with varying constrained layer damping coating configurations under explosive loading, the one-quarter finite element model was developed to match the experimental setup. The diagram of the finite element model is illustrated in Figure 13. The air-domain mesh dimensions were set to 155 mm × 155 mm × 70 mm. The Arbitrary Lagrangian–Eulerian (ALE) formulation was employed to model both the air and explosive components, whereas Lagrangian elements were utilized for the polyurea coating, concrete, and steel frame. Symmetry boundary conditions were applied along the symmetric planes, while non-reflecting boundary conditions were imposed on the remaining boundaries of the air domain to minimize wave reflections. The explosive charge was embedded within the air domain using the *INITIAL_VOLUME_FRACTION_GEOMETRY keyword. Since no debonding between the steel plate and polyurea was observed in the explosion tests, the steel plate and Q413t layer, as well as the Q413t and FPU-1 layers, were connected via shared nodes in the simulation to ensure full interfacial adhesion.

### 5.2. Material Model

In the model, the steel plate substrate was represented using the *MAT_PLASTIC_KINEMATIC material model to capture its mechanical behavior. The corresponding material parameters are summarized in Table 4.

The Q413t viscoelastic-damping polyurea (density (*ρ*) = 0.98 g/cm^3^; elastic modulus (*E*) = 1.82 MPa; Poisson’s ratio (*v*) = 0.4) and FPU-1 flexible polyurea (density (*ρ*) = 1.02 g/cm^3^; elastic modulus (*E*) = 35.73 MPa; Poisson’s ratio (*v*) = 0.4) were modeled by *MAT_PLASTICITY_POLYMER [22]. The *σ*_T_–*ε*_r_ curves at different strain rates are shown in Figure 4 and Figure 6.

The air was modeled using the *MAT_NULL material model in conjunction with the linear equation of state *EOS_LINEAR_POLYNOMIAL. The equation of state is detailed in Equation (1), and the corresponding material parameters are summarized in Table 5:

(1) $$p=C_{0}+C_{1}u+C_{2}u^{2}+C_{3}u^{3}+\left(C_{4}+C_{5}u+C_{6}u^{2}\right)e$$

where *e* is the internal energy in the ideal state; *u* is the relative volume; *C*_0_~*C*_6_ are the calculated equation coefficients.

The constitutive behavior of the explosive was modeled using *MAT_HIGH_EXPLOSIVE_BURN, and its equation of state was characterized by *EOS_JWL, which describes the pressure of the detonation products. The mathematical expression is provided in Equation (2), and the corresponding material parameters for the TNT are summarized in Table 6:

(2) $$p=A\left(1-\frac{\omega }{R_{1}V}\right)e^{-R_{1}V}+B\left(1-\frac{\omega }{R_{2}V}\right)e^{-R_{2}V}+\frac{\omega E}{V}$$

In the equation, *p* denotes the pressure of the detonation products, *V* represents the relative volume, and *A*, *B*, *R*_1_, *R*_2_, and ω are material constants. The first term on the right-hand side predominantly governs the behavior in the high-pressure regime, the second term is dominant in the medium-pressure range, and the third term accounts for the low-pressure behavior.

According to the observations from the on-site explosion tests, the steel frame exhibited no significant displacement or deformation. Consequently, it was modeled as a fully constrained rigid body in the simulation to improve the computational efficiency.

### 5.3. Structural Deformation Verification

**Figure 14:**
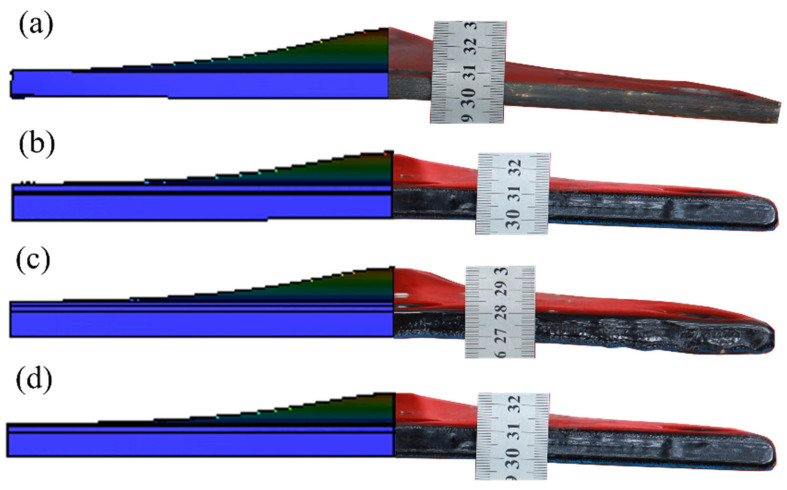
Comparison of deformation conditions between simulation results and explosion tests: (**a**) UC; (**b**) CLD1; (**c**) CLD2; (**d**) CLD3.

To validate the accuracy of the finite element model, the structural deformation observed in the explosion test is compared with the simulation results. Figure 14 illustrates the deformation comparison of the target plate between the experimental and numerical results. The simulation reveals a noticeable bulge in the central region of the specimen, which aligns well with the deformation observed in the actual explosion test.

Table 7 lists the displacements at the center points of the plates obtained from both the explosion test and the simulation. As shown in Table 7, the inaccuracies between the final displacement measured in the test and that predicted by the simulation remain below 8.7% across all four plates. These results indicate that the simulation outcomes are in good agreement with the experimental data, thereby validating the accuracy of the finite element model.

## 6. Simulation Results and Analysis

### 6.1. Response Process Analysis

This section investigates the protective performance of coating on steel plates by analyzing the dynamic responses on the back blast faces of the polyurea–steel composite plates, based on the established finite element models.

**Figure 15:**
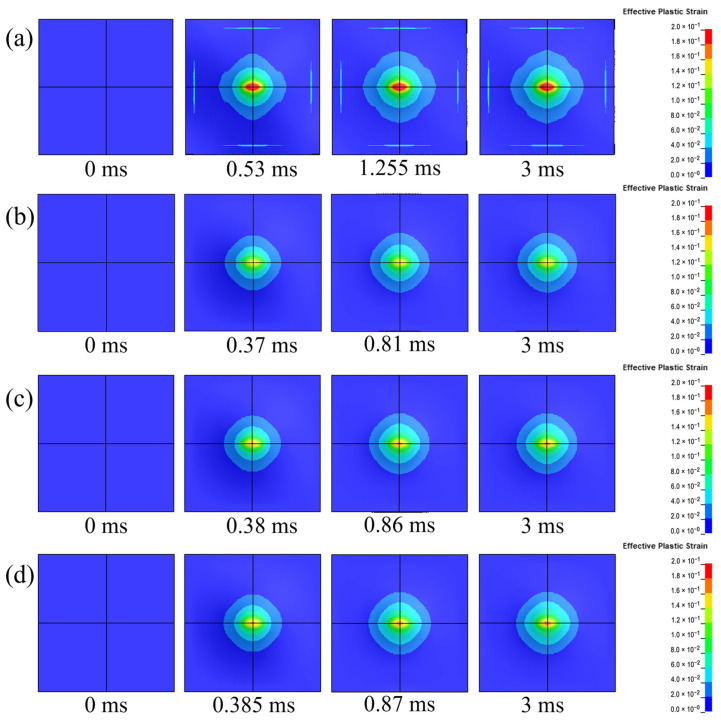
Effective plastic strain evolutions of steel plates: (**a**) UC; (**b**) CLD-1; (**c**) CLD-2; (**d**) CLD-3.

**Figure 16:**
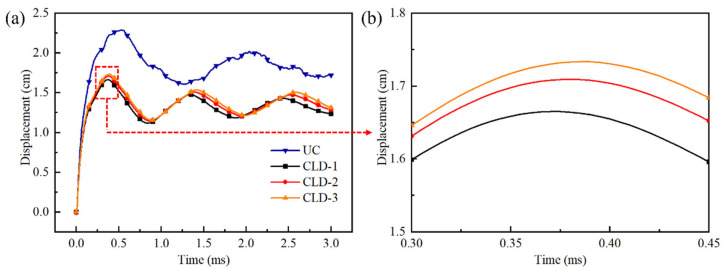
Displacement time history curves: (**a**) 0~3 ms; (**b**) 0.3 ms~0.45 ms.

Figure 15 presents the strain distribution contours of the steel layers on the blast side for the four plates. The figure includes snapshots at four key time points: the initial moment (t = 0 ms), the onset of rebound, the peak of the first rebound, and the final calculation time (t = 3 ms). Overall, the uncoated steel plate exhibits a significantly greater degree of deformation and a larger strain area (Figure 15a) compared to the polyurea–steel composite plates (Figure 15b–d). Temporally, following the initial rebound, the strain in all four plates stabilized, indicating that the majority of deformation occurred within a very brief period immediately after the explosion and not during the subsequent vibrational phase. In comparison with the uncoated plate, the polyurea–steel composite plates demonstrate a notably delayed initial rebound. When analyzed in conjunction with the displacement time history curve (Figure 16), it becomes evident that applying the constrained layer damping coating to the back blast face of the steel plate can effectively mitigate deformation.

Through magnification of the displacement–time history curves for the three types of polyurea–steel composite plates, it is evident that CLD1 exhibits the smallest peak displacement. A comparative analysis of the effective plastic strains in the three polyurea–steel composite plates at 3 ms reveals that, among the three configurations, only CLD1 shows no red regions in the effective plastic strain contour of the central steel plate area, indicating that CLD1 underwent the least deformation.

**Figure 17:**
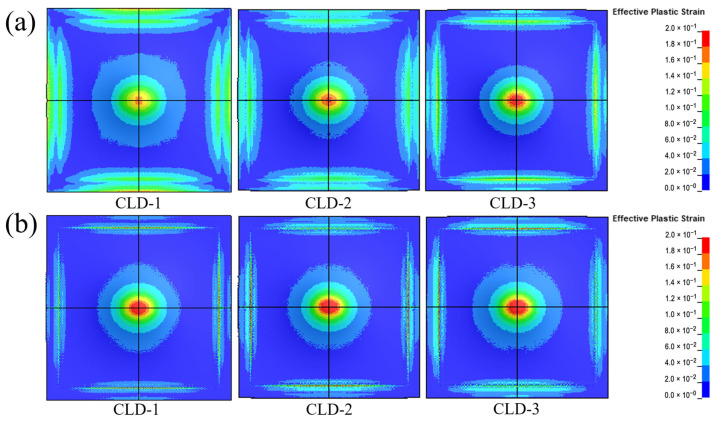
The effective plastic strain nephograms of (**a**) Q413t (damping layer) and (**b**) FPU-1 (constraining layer).

To investigate the dynamic responses of polyurea–steel composite plates with varying thickness ratios, the effective plastic strain nephograms of Q413t (damping layer) and FPU-1 (constraining layer) in three types of polyurea–steel composite plates (Figure 17) are examined. For Q413t, its strain area was notably influenced by the coating thickness: a thicker damping layer resulted in a smaller overall strain area. Conversely, the area of the maximum strain region (indicated by the red zones in Figure 17) increased proportionally with the damping layer thickness. This suggests that, in a constrained layer damping coating structure, the damping layer also functions to disperse concentrated loads. Based on the mechanical properties of Q413t at different strain rates discussed in Section 3.1, this load dispersion effect can be attributed to two factors. First, Q413t possesses low strength and high elongation, making it highly deformable and capable of distributing applied loads. However, as the damping layer thickens, its deformation resistance increases, which, in turn, reduces its ability to disperse loads. Second, efficient load transfer depends strongly on interfacial adhesion between the damping layer and the substrate. Failure of this adhesive bond may lead to fracture of the damping layer.

In contrast to the damping layer, the primary function of the constraining layer polyurea is to restrict structural deformation. Due to its superior mechanical properties, FPU-1 exhibits limited load diffusion. As shown in the strain cloud diagrams of Figure 17b, despite variations in the constraining layer thicknesses among the three composite coatings, the strain distributions remain relatively similar. However, in CLD1, where the damping layer is thinner, the stress dispersion effect is more effective, resulting in a smaller strain area. From the perspective of mechanical strength in constrained layer damping composite coatings, CLD1 benefits from a thicker constraint layer, which enhances the overall structural rigidity and provides the most effective constraint on deformation. Therefore, in the design of constrained-damping coatings, a thinner damping layer combined with a thicker constraining layer offers superior protection against blast-induced structural damage.

### 6.2. Composite Structure Energy Analysis

**Figure 18:**
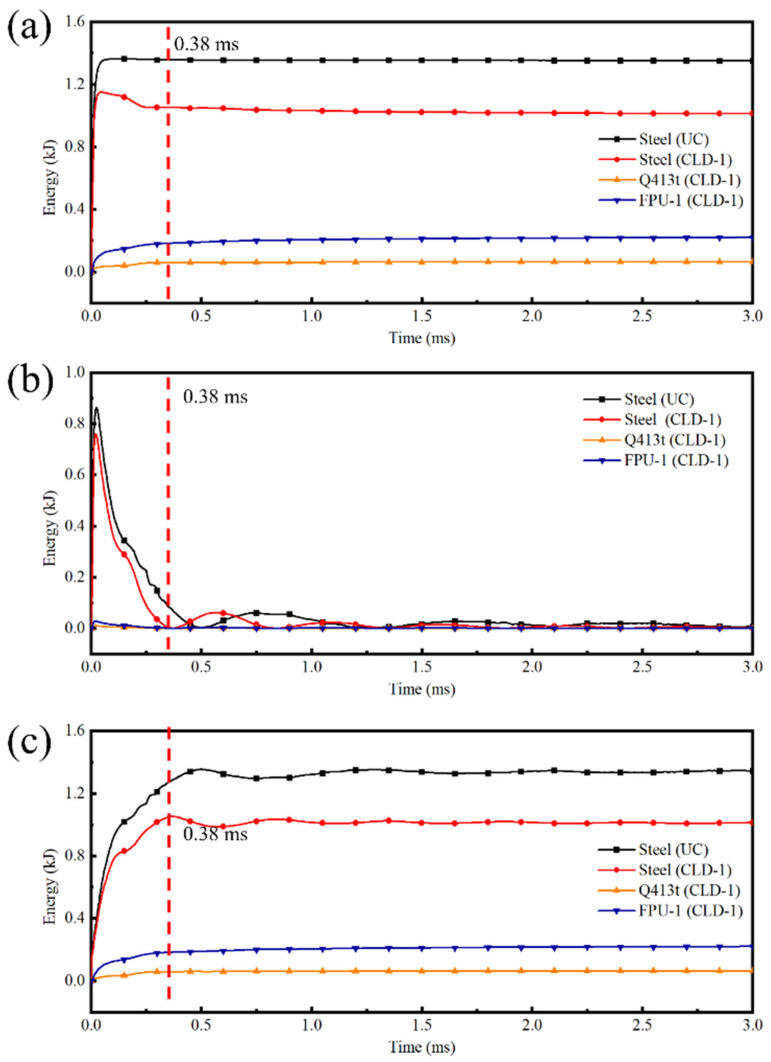
Energy curves of the unprotected steel plate and CLD1: (**a**) total energy; (**b**) kinetic energy; (**c**) internal energy.

To investigate the energy variation in polyurea–steel composite plates, this section analyzes the blast mitigation mechanism of coated protective steel plates using CLD-1 as a representative example. Figure 18 illustrates the energy absorption curves of individual materials within CLD-1, with the UC plate serving as the control test.

Based on the energy variations (Figure 18) and displacement characteristics (Figure 16), the dynamic response of CLD-1 can be clearly divided into two distinct stages: the tensile deformation stage (0–0.38 ms) and the subsequent vibration stage (0.38–3 ms). During the first stage, the polyurea–steel composite plate undergoes predominantly positive deformation, whereas in the second stage, it exhibits oscillatory behavior.

In the initial deformation stage, the total energy of the steel component in CLD-1 rapidly increases and then decreases (Figure 18a), while in the UC plate, the steel energy rises and remains nearly constant. By analyzing both the kinetic energy curve (Figure 18b) and internal energy curve (Figure 18c), it is evident that as the kinetic energy of the steel plate diminishes, the internal energy of the polyurea layer increases significantly. This indicates that the reduction in steel energy is due to the polyurea layer absorbing part of the steel’s kinetic energy through its own deformation. Furthermore, as shown in Figure 18a, the peak total energy of the steel in CLD-1 is 1.17 kJ, which is 13.97% lower than that of the UC plate (1.36 kJ), demonstrating that the presence of the polyurea layer directly reduces the total energy absorbed by the steel plate.

In the second stage, the total energies of both the steel and polyurea remain nearly constant (Figure 18a), although energy conversion between kinetic energy (Figure 18b) and internal energy (Figure 18c) continues within the steel component. This suggests that during the vibration phase, the polyurea layer has minimal influence on the overall energy state of the steel.

These findings indicate that the polyurea layer contributes to energy reduction in the steel plate through two mechanisms: direct energy absorption and suppression of structural deformation, thereby providing effective protection against blast loading.

To investigate the effect of coatings with different thickness ratios on the protective performance of the steel plate, Table 8 summarizes the final absorbed energies of various polyurea–steel composite plates and an uncoated plate. As shown in Table 8, the energies absorbed by the steel component in all three coated configurations are lower than that in the UC plate. This, together with the effective plastic strain evolution (Figure 15) and displacement curve (Figure 16), demonstrates that the polyurea coating effectively absorbs blast energy and mitigates both the extent of the damage and deformation of the steel plate.

As is evident from Figure 18, during the rebound process, the internal energy of the polyurea coating does not exhibit any attenuation. In contrast, there is a notable decrease in the kinetic energy of the steel plate. This phenomenon suggests that the rebound of the polyurea–steel composite plate is predominantly attributed to the release of the steel’s elastic energy. Moreover, the internal energy of the polyurea coating is dissipated as viscoelastic energy. As shown in Table 8, a comparative analysis of plates with varying thickness ratios of polyurea coating reveals that CLD-1 exhibits the lowest energy absorption capacity in the steel plate and the highest viscoelastic dissipation energy within the polyurea layer. Conversely, CLD-3 demonstrates the highest energy absorption by the steel plate and the lowest viscoelastic dissipation energy in the polyurea coating. Considering the energy absorption capacities of Q413t and FPU-1, it is evident that FPU-1 has a higher energy absorption per unit surface density than Q413t. Consequently, in CLD-1, which features the thickest FPU-1 layer (3 cm), the steel layer absorbs the least amount of energy. This also explains why CLD-1 exhibits the smallest center point displacement. It should be emphasized, however, that the energy discussed here specifically refers to the strain energy resulting from material deformation, as opposed to the energy associated with molecular chain segment motion described in Section 4.2.

Additionally, the constrained layer damping coating structure inherently functions as a specialized energy-absorbing configuration. When subjected to explosive loads, the strength mismatch between the constraining layer and the damping layer induces relative displacement, which dissipates explosive energy through shear deformation. During the application of the explosive load, the polyurea–steel composite plate vibrates, causing the composite coating to undergo reciprocating motion that enhances the damping layer’s capacity for energy absorption. Furthermore, in the case of back blast face coatings, the relative displacement between the steel plate and the constraining layer not only improves the damping efficiency of the damping layer but also reduces, to some extent, the strain rate experienced by the constraining layer under the explosive load. The synergistic interaction between the constraining layer and the damping layer enables the composite coating to provide effective blast protection for the steel plate.

## 7. Conclusions

Polyurea, as a high-performance protective material, has shown great potential in enhancing the blast resistance of metallic structures and has undergone rapid development in recent years. However, most of the existing studies have focused on single-type polyurea. Expanding on previous research into the effects of polyurea’s damping behavior on structural blast protection, this study adopts a constrained layer damping coating configuration to improve the blast resistance of steel plates. The mechanical properties of the viscoelastic damping polyurea and flexible polyurea under various strain-rate conditions, as well as their damping behaviors under different loading frequencies, are systematically analyzed. Furthermore, the protective mechanisms and design principles of the damping and constraining layers under explosive load are elaborated. The main conclusions are summarized as follows:

1. Q413t shows a pronounced strain-rate effect under intermediate strain rates. At a strain rate of 770.88 s^−1^, its maximum true stress increased by 104%. In contrast, FPU-1 experienced a significant increase in maximum engineering stress due to the strain-rate effect, but its breaking elongation decreased notably. Specifically, at a strain rate of 759.12 s^−1^, the breaking elongation of FPU-1 decreased by 49% compared to that at 0.1 s^−1^, resulting in a relatively stable maximum true stress.
2. Q413t exhibits a superior damping performance over a broad effective-damping frequency range (0–10^4^ Hz), whereas FPU-1 demonstrates effective damping within a narrower frequency range of 10^4^–10^5^ Hz. When combined into a constrained damping composite coating, the resulting structure achieves an extended effective damping frequency range, which enhances the coating’s ability to absorb and mitigate external loads.
3. Both explosion tests and finite element simulations confirm that the constrained-damping composite coating applied to the back blast faces of steel plates significantly improves their blast resistance. In this structure, the damping layer mitigates structural damage by dispersing the explosive load and dissipating energy, while the constraining layer suppresses deformation due to its higher mechanical strength. Additionally, the relative displacement between the two layers contributes to an overall enhancement of the system’s damping performance. Integrating experimental and simulation results with the protective characteristics of the two materials, it is determined that the optimal thickness ratio of the damping layer to the constraint layer is 1:3. The constrained layer damping coating synergistically enhances mechanical strength and energy dissipation, effectively reducing structural deformation and demonstrating promising potential for engineering applications.

## Figures and Tables

**Table 1:** Main components.

Components	Relative Molecular Mass	Functionality	Manufacturer
Methylene diphenyl diisocyanate (MDI-50)	250	2	Wanhua Chemical Group Co., Ltd., Yantai, China
Polytetramethyleneoxide-di-p-aminobenzoate (P-1000)	1000	2	Suzhou Xiangyuan New Materials Co., Ltd., Suzhou, China
3-Chloro-4,4′-diaminodiphenylmethane (ML-400)	244	2	Suzhou Xiangyuan New Materials Co., Ltd., Suzhou, China
Q413t viscoelastic damping polyurea (Q413t) component A	-	-	Qingdao Shamu Advanced Material Co., Ltd., Qingdao, China
Q413t viscoelastic damping polyurea (Q413t) component B			Qingdao Shamu Advanced Material Co., Ltd., Qingdao, China

**Table 2:** Design and dimensional specifications of polyurea–steel composite plates.

I.D.	Thickness of Damping Layer (mm)	Thickness of Constraining Layer (mm)	Schematic Diagram
UC	-	-	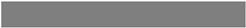
CLD-1	1	3	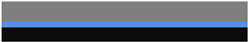
CLD-2	2	2	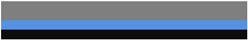
CLD-3	3	1	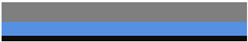

Note: In Table 2, 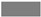 represents the steel plate; 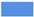 represents the damping layer; 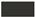 represents the constraining layer.

**Table 3:** Mechanical properties of Q413t and FPU-1 at low strain rates.

Type	Strain (s^−1^)	Elastic Modulus (MPa)	Tangent Modulus (MPa)	Tensile Strength (MPa)	Elongation (%)
Q413t	0.001	1.06 ± 0.01	0.38 ± 0.01	0.51 ± 0.01	≥73
	0.01	1.52 ± 0.01	0.41 ± 0.01	2.89 ± 0.01	≥445
	0.1	1.82 ± 0.03	0.41 ± 0.01	3.33 ± 0.02	≥467
FPU-1	0.001	23.66 ± 0.07	1.58 ± 0.01	17.62 ± 0.64	≥420
	0.01	27.78 ± 0.24	2.39 ± 0.01	20.24 ± 0.35	≥381
	0.1	35.73 ± 0.95	2.62 ± 0.01	21.28 ± 1.02	≥368

**Table 4:** Material parameters of steel.

***ρ* (g/cm^3^)**	***E* (GPa)**	***ν***	***σ_0_*** **(GPa)**	***E_t_* (GPa)**
7.85	210	0.3	0.235	2.1

**Table 5:** Material parameters of air.

*ρ* (g·cm^−3^)	*C*_0_–*C*_3_	*C*_4_	*C*_5_	*e* (J·cm^−3^)
0.00129	0	0.4	0.4	2.5 × 10^5^

**Table 6:** Material parameters of TNT.

*ρ* (g·cm^−3^)	*D* (m·s^−1^)	*P*_CJ_ (GPa)	*A* (GPa)	*B* (GPa)
1.58	6880	19.4	307	3.898

Note: *D* is the detonation velocity of the TNT. *P*_CJ_ is the Chapman–Jouguet pressure.

**Table 7:** Comparison of final displacement results between experimental tests and simulations.

I.D.	Experimental Results (cm)	Numerical Simulation (cm)	Inaccuracies (%)
UC	1.90	1.86	−2.1
CLD-1	1.23	1.31	6.5
CLD-2	1.37	1.34	−2.1
CLD-3	1.49	1.36	−8.7

**Table 8:** Absorbed energies of different structural configurations.

I.D.	Total Final Absorbed Energy (J)			Final Kinetic Energy (J)			Final Internal Energy (J)			Viscoelastic Dissipation of Constrained Layer Damping Coating (J)
	Steel	Q413t	FPU-1	Steel	Q413t	FPU-1	Steel	Q413t	FPU-1	
UC	1351			6.27			1344			
CLD-1	1012	64	219	0.10	0.0012	0.0036	1012	64	219	283
CLD-2	1034	102	162	2.76	0.069	0.069	1032	102	162	264
CLD-3	1060	143	97	6.10	0.23	0.077	1054	143	97	240

## Data Availability

The original contributions presented in this study are included in the article. Further inquiries can be directed to the corresponding author.
